# Is lecture dead? A preliminary study of medical students’ evaluation of teaching methods in the preclinical curriculum

**DOI:** 10.5116/ijme.59b9.5f40

**Published:** 2017-09-22

**Authors:** Anne Zinski, Kristina T.C. Panizzi Woodley Blackwell, F. Mike Belue, William S. Brooks

**Affiliations:** 1 Department of Medical Education, University of Alabama at Birmingham, Birmingham, AL, USA; 2 Department of Cell, Developmental and Integrative Biology, University of Alabama at Birmingham, Birmingham, AL, USA

**Keywords:** Undergraduate medical education, instructional methods, collaborative learning, case-based learning, team-based learning, lecture, simulation, laboratory, patient presentation

## Abstract

**Objectives:**

To investigate medical students’ perceptions of lecture and non-lecture-based instructional methods and compare preferences for use and quantity of each during preclinical training.

**Methods:**

We administered a survey to first- and second-year undergraduate medical students at the University of Alabama School of Medicine in Birmingham, Alabama, USA aimed to evaluate preferred instructional methods.  Using a cross-sectional study design, Likert scale ratings and student rankings were used to determine preferences among lecture, laboratory, team-based learning, simulation, small group case-based learning, large group case-based learning, patient presentation, and peer teaching. We calculated mean ratings for each instructional method and used chi-square tests to compare proportions of first- and second-year cohorts who ranked each in their top 5 preferred methods.

**Results:**

Among participating students, lecture (M=3.6, SD=1.0), team based learning (M=4.2, SD=1.0), simulation (M=4.0, SD=1.0), small group case-based learning (M=3.8, SD=1.0), laboratory (M=3.6, SD=1.0), and patient presentation (M=3.8, SD=0.9) received higher scores than other instructional methods. Overall, second-year students ranked lecture lower (χ^2^_(1, N=120)_ =16.33, p<0.0001) and patient presentation higher (χ^2^_(1, N=120)_ =3.75, p=0.05) than first-year students.

**Conclusions:**

While clinically-oriented teaching methods were preferred by second-year medical students, lecture-based instruction was popular among first-year students. Results warrant further investigation to determine the ideal balance of didactic methods in undergraduate medical education, specifically curricula that employ patient-oriented instruction during the second preclinical year.

## Introduction

The premise of undergraduate medical education is the scaffolding of knowledge in the basic and clinical sciences with the goal of producing competent, well-rounded physicians who are engaged in patient care. The preclinical years are recognized as a formative time to instill lifelong learning and skills that will prepare students for practicing in the clinical environment.^1^ Though traditional lectures are an acceptable instructional method for conveying knowledge and remain the predominant mode of teaching in US medical schools,^2^ there has been a greater emphasis in recent years to encourage self-directed learning,^3^ non-lecture-based instructional methods,^4^ patient-oriented learning,^5^ and student-centric individualized and team learning in the preclinical curriculum.^6^^,^^7^ This realignment of instructional methods has been encouraged by the Liaison Committee on Medical Education (LCME).

The shift from a traditional, lecture-based teaching model to extensive incorporation of non-lecture-based instructional strategies presents a number of challenges to medical educators. First, a variety of instructional methods are available from which to choose, and educators and students may lack familiarity or comfort with their utilization, particularly when content is not delivered in the same manner or covered to the same extent as lecture-based approaches.^4^^,^^8^^,^^9^ Some instructional strategies require supplementary resources including space, time, or an increased number of faculty or staff, which can place additional burden on the medical school.^10^ Furthermore, with the available resources for accessing emerging basic science and clinical knowledge, educators may be unsure of students’ abilities to independently seek, sort, and prioritize this information appropriately.

Recent literature has compared and contrasted the utility of specific instructional methods versus lecture, with many studies focused on evaluating learning outcomes and student satisfaction with problem-based learning,^11^^,^^12^ team-based learning,^13^^-^^15^ and simulation.^16^^,^^17^ Similarly, learner engagement during lecture, problem-based learning, and team learning have been examined.^18^ Far fewer studies compare students’ perceptions of multiple instructional methods across the two preclinical years, though multimodal instruction has been advocated.^19^ Furthermore, despite these changes in medical education, few studies have examined millennial generation learners’ perceptions of didactic instruction in medical school. Millennial learners employ digital methodology and are team-oriented, suggesting preferences for collaborative and technology based learning approaches;^20^ however, Tsang and Harris posit that medical students favor lecture and passive teaching methods for learning.^21^

Incorporating students’ perspectives of instructional method is critical for accommodating preferred learning styles and the evolving technological landscape. Current students are a primary stakeholder for determining optimal distribution of methodologies. While end-of-course student evaluations are traditionally used to gauge the quality of various methods within a course, this information is context-dependent. Here, we sought to examine students’ attitudes toward methods of teaching through a comprehensive evaluation.

The purpose of this study is to evaluate students’ perspectives on lecture and non-lecture-based instructional methods currently in use in the University of Alabama School of Medicine (UASOM) preclinical curriculum. Study objectives are to determine students’ ratings of instructional methods, collect students’ ranking of each preferred instructional method, and gauge students’ preference for hours of exposure to each method in the preclinical medical curriculum. We hypothesize that medical students prefer non-lecture-based instructional methods over didactic lecture.

## Methods

Medical students’ perceptions of eight instructional methods used in the preclinical curriculum were evaluated at the University of Alabama School of Medicine (UASOM) through a cross-sectional study design. During a quality improvement initiative in February 2016, we invited first-year (MS1) and second-year (MS2) undergraduate medical students via email to participate in an anonymous survey to evaluate preclinical instruction at the UASOM. Students consented to participate via survey completion. The study was approved with exempt status for analysis of the existing dataset for research by the University of Alabama at Birmingham (UAB) Institutional Review Board for Human Use.

### Description of medical school

The UASOM is a multi-campus medical school with all students completing preclinical training (Years 1 and 2) at the Birmingham campus, followed by clinical training (Years 3 and 4) at the Birmingham campus or one of three regional campuses located in Huntsville, Montgomery, or Tuscaloosa, Alabama. Preclinical instruction at the UASOM is an integrated, single-pass organ system-based curriculum, which has been previously described.^22^ Year 1 includes Patient, Doctor, and Society; Fundamentals of Medicine; Cardiovascular; Pulmonary; Gastrointestinal; Renal; and Introduction to Clinical Medicine I. Year 2 is composed of Musculoskeletal and Skin, Neurosciences, Hematology and Oncology, Endocrine, Reproduction, and Introduction to Clinical Medicine II (Appendix 1). First- and second-year medical students were surveyed following completion of the first course in their second respective semesters.

### Data collection method

An invitation to participate was distributed via email to all first-year and second-year medical students in February 2016 with a link to an online survey to investigate preferred instructional methods at the UASOM. Ten instructional methods were originally included: lecture, team-based learning (TBL), laboratory, small group case-based learning (CBL), large group case-based learning, patient presentation, simulation, peer teaching, reflection, and group project/presentation. Reflection and group project/presentation were later removed from analysis due to low usage in the UASOM preclinical curriculum as measured by contact time. In addition to the instructional method name, an adapted definition from the MedBiquitous Curriculum Inventory Standardized Vocabulary^23^ was provided for each term in the survey (Appendix 2). The survey tool consisted of 4 total items: 1 item in which students were asked to rate each named instructional method using a 5-point Likert scale (Alpha=0.66); 1 item in which students were asked to report whether they preferred more, less, or the same exposure to each instructional method (Alpha=0.48); 1 item in which students were asked to select and rank their five preferred instructional methods; and finally, 1 additional item that allowed students to comment on each instructional method in a free response format. The survey was administered anonymously. Each student identified his/her year in medical school as a descriptor; no other demographic data was obtained.

### Contact hours by method

Contact hours of each instructional method were calculated from a central curriculum mapping software for courses in the 2015-16 and 2016-17 academic years and combined to determine the total instructional contact time for each year. The percentage of contact time for each method was determined by dividing the number of contact hours that each method was utilized by the total number of instructional contact hours in the preclinical curriculum. The focus of the study was on instruction within the basic science coursework, so the clinical skills courses were excluded. Endocrine and Reproduction courses were also excluded, as MS2 students had not yet completed these courses at the time of survey distribution. Contact hours devoted to course introductions, assessments, and review sessions were also excluded.

### Data analysis

Descriptive statistics were reported in the form of mean and standard deviation for Likert scale items or frequencies and proportions for rankings. Two-tailed student’s t-tests were used to compare students’ ratings of instructional methods by year (MS1 vs MS2). For analyzing rank order preferences of instructional methods, we calculated the proportion of MS1 and MS2 students who ranked each method in their top 5 preferred methods and documented the number of students who ranked each method as 1st, 2nd, 3rd, 4th, or 5th. We conducted χ^2^ tests to gauge differences in the proportions of students who ranked each method in the top 5.  Significance was set at p<0.05. Survey response data was exported to Microsoft Excel for storage, organization, and cleaning. All data analyses were generated in JMP Pro 13.1.0.

## Results

Fifty-five MS1 and 65 MS2 students participated in the survey, yielding 29% and 35% response rates, respectively.  No significant differences in the mean composite Medical College Admissions Test (MCAT) score or undergraduate grade point average existed between the student cohorts.  Team-based learning (M=4.2, SD=1.0) and simulation (M=4.0, SD=1.0) yielded the highest mean scores for preferred instructional methods (Table 1) among both groups, followed by patient presentation (M=3.8, SD=0.9), small group case-based learning (M=3.8, SD=1.0), lecture (M=3.6, SD=1.0), and laboratory (M=3.6, SD=1.0).  By cohort, MS2 students rated lecture (t_(__114)_=3.48, p=0.0007), small group CBL (t_(118)_=3.13, p=0.002), and laboratory (t_(117)_=2.86, p=0.005) significantly lower than their MS1 counterparts.  No significant differences were observed for TBL, large group CBL, simulation, patient presentation or peer teaching scores.

For the instructional methods ranking item (Table 2), 94.5% of MS1 students included lecture in their top 5 preferred methods, and significantly fewer (66.2%) MS2 students did likewise (χ^2^_(1, N=120)_ =16.33, p<0.0001). For patient presentation, 63.1% of MS2 students ranked this method in their top 5 preferred methods versus 45.5% of MS1 (χ^2^_(1, N=120)_ =3.75, p=0.05). No significant differences by cohort were observed for other instructional methods.

For contact hours, approximately half of student respondents indicated that they preferred more TBL (58.4%) and simulation (46.9%). Large proportions of students preferred the same amount of patient presentation (45.1%), small group CBL (41.6%), laboratory (70.2%), and lecture (61.4%). Students preferred less instructional contact hours for large group CBL (57.9%). For peer teaching, results were mixed, with 47.4% of respondents preferring less and 41.2% preferring the same contact hours.

Of the 801 total hours of preclinical instructional contact hours that were included, lecture was utilized most often (65%, 524 of 801), followed by laboratory (10%), small group CBL (8%), and large group CBL (7%).  TBL, patient presentations, peer teaching, and simulation each represented 1-3% of total contact time. ‘Other’ instructional time represented 3% of total contact time and included panel discussions and flipped classrooms, among others.

**Table 1:** Students' evaluation of eight instructional methods

Cohort	Lecture	Team-based learning	Small case-based learning	Large case-based learning	Laboratory	Simulation	Patient Presentation	Peer Teaching
	Mean(SD)	Mean(SD)	Mean(SD)	Mean(SD)	Mean(SD)	Mean(SD)	Mean(SD)	Mean(SD)
All	3.6(1.0)	4.2(1.0)	3.8(1.0)	2.9(1.0)	3.6(1.0)	4.0(1.0)	3.8(0.9)	3.0(1.0)
MS1	4.0(0.7)	4.4(0.8)	4.1(0.8)	3.0(0.9)	3.9(0.8)	4.1(0.9)	3.8(0.8)	3.2(0.9)
MS2	3.4(1.1)	4.0(1.2)	3.5(1.1)	2.8(1.2)	3.3(1.1)	3.8(1.0)	3.7(1.0)	2.8(1.1)
p	<0.01	0.08	<0.01	0.23	<0.01	0.12	0.56	0.07

**Table 2:** Students' ranking of instructional methods as 1st, 2nd, 3rd, 4th, or 5th with percentage of students who included each in their top 5 preferred methods

Cohort	Ranking	Lecture	Team-based learning	Small case-based learning	Large case-based learning	Laboratory	Simulation	Patient Presentation	Peer Teaching
MS1 (N=55)	1	19	18	6	0	1	8	3	0
	2	5	11	14	1	9	10	3	2
	3	12	11	15	2	6	3	3	2
	4	10	6	7	6	9	7	6	1
	5	6	4	2	4	10	8	10	5
	n(%)	52(94.5)	50(90.9)	44(80.0)	13(23.6)	35(63.3)	36(65.5)	25(45.5)	10(18.2)
										MS2 (N=65)	1	22	17	8	0	1	10	5	1
2	7	24	7	0	8	6	10	1		
3	9	4	12	5	8	12	9	2		
4	3	8	11	7	4	14	10	2		
5	2	4	10	9	14	5	7	6		
n(%)	43(66.2)	57(87.7)	48(73.8)	21(32.3)	35(53.8)	47(72.3)	41(63.1)	12(18.5)		
	p	<0.01	0.57	0.43	0.29	0.28	0.42	0.05	0.97

## Discussion

The medical education literature is replete with studies highlighting the utility of a variety of instructional methods. Medical educators and administrators must designate the highest quality instructional methods to teach the knowledge, skills, and attitudes essential to clinical practice.^4^^,^^24^ The complexity of these decisions is compounded by the variety of curricular structures currently used in undergraduate medical training^25^ as well as the diverse learning styles present amongst students,^26^^,^^27^ which prompted the present investigation.

While we hypothesized that lecture would be a less preferred instructional method, as it is categorized as passive learning^28^ and may offer lesser knowledge retention rates,^28^ students ranked lecture highly amongst the methods evaluated. This was particularly evident for first-year medical students, a finding similar to that of Tsang and Harris.^21^ There are a number of possible explanations for these findings. First-year medical students may be more comfortable with traditional, lecture-based instructional methods following undergraduate training. Lecture is also an efficient method for delivering sizeable amounts of factual information to a large group of learners and was shown to be more beneficial than case-based learning for examination preparation.^29^ Another possible explanation for students’ evaluations of lecture methodology is the availability of lectures online for remote student viewing outside of allotted lecture hours.  Despite the potential for differences in knowledge retention, students may value the Table 1. Students' evaluation of eight instructional methods flexibility provided by optional lecture attendance and the ability to watch recorded lectures multiple times, at varying speeds and at the location of their choice.

A noteworthy finding that warrants further exploration is the significantly lower proportion of MS2 students who ranked lecture in their top 5 methods versus first year students. Additionally, a significantly higher proportion of MS2 students ranked patient presentation into the top 5, and a higher number of MS2 students chose simulation as a top 5 instructional method, though not statistically significant. A potential interpretation for this finding may reflect the shifting away from the delivery of factual information via lecture in their first year towards clinical or case-focused learning in subsequent years. It has been shown that students’ clinical reasoning skills increase during the preclinical years^30^ and that clinical application with patient contact contributes to the development of these skills.^5^ Following the acquisition of a fundamental biomedical science knowledge base, students increase exposure to clinical medicine and grow adept at applying knowledge to diagnose clinical problems; this may contribute to a growing preference for clinically focused instruction, such as patient presentation and simulation.

Beyond lecture, other highly ranked instructional methods amongst MS1 students included TBL, small group CBL, simulation, and laboratory. Interestingly, each of these active learning methods involves a collaborative learning component.  Additionally, student groups which participate in these teaching sessions have the same roster throughout preclinical training at the UASOM. The utility of and learner preferences for active learning methods involving learner-to-learner interaction have been well documented.^14^^,^^31^^,^^32^ Furthermore, the development of teamwork skills is critical for practicing physicians.^6^^,^^33^ The use of these methods in preclinical education may warrant further investigation for future generations of medical school learners.

Large group CBL was rated lower and ranked poorly by a higher proportion of MS1 and MS2 students, while small group CBL was preferred by more students by rank and rating. Both methods provide the opportunity for students to learn basic science content within a clinical framework through guided discussions in a structured setting,^34^and each method was utilized frequently in the UASOM preclinical curriculum. One factor that may be associated with the rating discrepancy is that students may feel more comfortable interacting with and receiving personalized feedback within a smaller group of peers. Small group CBL, similar to TBL, offers students the opportunity to explore areas of uncertainty and ask questions in a safe learning environment,^35^^,^^36^ whereas asking questions in a larger group setting may be intimidating. Additionally, unlike large group CBL in which students’ participation is largely independent, small group CBL and TBL place much of the learning focus on the combined effort of the group.^37^

While it is essential to weigh student preferences for instructional methods, there are a myriad of components that contribute to course planning decisions, including learner and instructor characteristics, the intended learning outcomes, and cognitive load imposed by instructional design.^26^^,^^38^^,^^39^  Preferred learning styles vary within cohorts of medical students; similarly, educators employ teaching styles with which they are most comfortable and adept. While it is appropriate to consider a balance of students’ and instructors’ preferences for learning style and teaching style, respectively, Vaughn and Baker posit that teaching/learning style mismatches are necessary for optimal learning.^26^ Placing students into a learning environment with a certain degree of tension prevents students from becoming bored and expands their ability to utilize non-dominant learning styles and flexibility in learning.^39^^, ^^40^ 

Intended learning outcomes or specific curricula may further inform the selection of instructional methods. For example, while simulation may be the optimal method to reinforce positive attitudes toward interprofessional collaboration, a lecture or patient presentation may be less useful in this area. Educators must also consider the impact of instructional methodology choice on both cognitive load and practical effort. For instance, instructional sessions that require a large volume of pre-class work, such as TBL, may be most useful when strategically scheduled in the curriculum to reduce student burden. Furthermore, faculty must be aware of the cognitive load imposed on learners by various instructional methods and take steps to keep this load manageable for optimal learning.

This study has several limitations. First, the response rates (29% for MS1 and 35% for MS2) were low and may not be representative of the entire student body. Potential explanations for the low response rates include a lack of incentive or survey fatigue from multiple student surveys and regular course evaluations. Additionally, only eight instructional methods available to educators were investigated, and the survey did not ask about specific exposure to each method as part of the preclinical curriculum. Notable omissions include problem-based learning (PBL), concept mapping, and workshop, among others. All data presented reflect students’ evaluations of instructional methods in the context of the preclinical curriculum of one medical school; students’ perceptions about instructional methods were likely impacted by type of curriculum, prior exposure and experience with each method, learning style preferences, and the quality of the faculty instructors’ implementation of each method, among others. Furthermore, these results are specific to preclinical undergraduate medical education and may lack application to clinical undergraduate medical education and graduate medical education.

## Conclusions

Lecture, TBL, small group CBL, laboratory, simulation, and patient presentation were rated highly by students in the preclinical curriculum. Second-year students rated patient presentation highly, and differed from first year students with respect to ratings for lecture. As medical educators map and implement their teaching methods for basic science objectives in preclinical education, this study supports the inclusion of lecture-based instruction. However, faculty and administrators may consider purposefully shifting lecture-based content in year 1 to a clinical-based instructional approach in year 2 with appropriate learning support. Future investigations of instructional methods such as TBL, laboratory, and small group CBL across the MS1 and MS2 years with a gradual reduction of lecture for increased patient presentation and simulation are warranted, as well as qualitative inquiry to decipher the precise utility of each instructional method.

### Acknowledgements

The authors would like to thank Dr Ashutosh Tamhane for his thoughtful consideration and suggestions for data analysis.  We also thank the UASOM Medical Education Research Interest Group for their helpful discussions of the data in this manuscript.

### Conflict of Interest

The authors declare that they have no conflict of interest.

## Supplementary material

Supplementary file 1 Appendix 1. UASOM Preclinical Curriculum

Supplementary file 2 Appendix 2. Definitions of 8 instructional methods provided in survey
