# Supplementary material for: Is lecture dead? A preliminary study of medical students’ evaluation of teaching methods in the preclinical curriculum
Source: Int J Med Educ. 2017 Sep 22;8:326–33. doi: 10.5116/ijme.59b9.5f40 (PMC5699863; doi:10.5116/ijme.59b9.5f40)

Appendix 1

UASOM Preclinical Curriculum

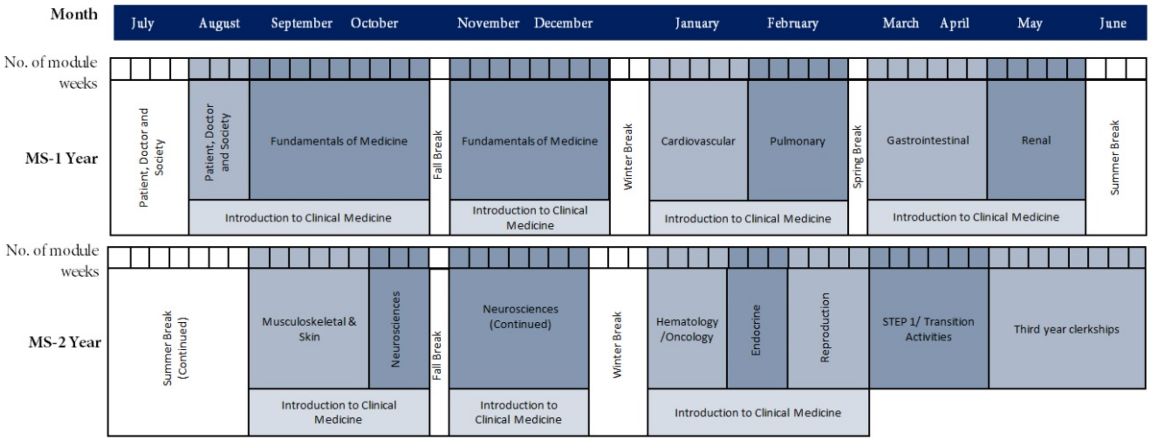

Supplement: Supplementary file 1 — Appendix 1. UASOM Preclinical Curriculum [file ijme-8-326-S1.pdf]
