# Supplementary material for: Is lecture dead? A preliminary study of medical students’ evaluation of teaching methods in the preclinical curriculum
Source: Int J Med Educ. 2017 Sep 22;8:326–33. doi: 10.5116/ijme.59b9.5f40 (PMC5699863; doi:10.5116/ijme.59b9.5f40)
Supplement: Supplementary file 2 — Appendix 2. Definitions of 8 instructional methods provided in survey [file ijme-8-326-S2.pdf]

Appendix 2

Definitions of 8 instructional methods provided in survey[23]

Lecture – an instruction by a speaker before a large group of learners

Team-based Learning (TBL) – a form of collaborative learning that follows a specific sequence of individual work and group work; engages learners in activities within a small group that works independently in classes with high learner-faculty ratios

Small Group Case-based Learning (CBL) –the use of patient cases to stimulate discussion, questioning, problem solving, and reasoning on issues pertaining to the basic sciences and clinical disciplines [less than 12 learners]

Large Group Case-based Learning (CBL) –the use of patient cases to stimulate discussion, questioning, problem solving, and reasoning on issues pertaining to the basic sciences and clinical disciplines [greater than 12 learners]

Laboratory – hands-on or simulated exercises including anatomy lab, histology lab, and microbiology lab

Simulation – a method used to replace or amplify real patient encounters with scenarios designed to replicate real health care situations, using lifelike mannequins, physical models, standardized patients, or computers

Patient Presentation by Faculty – a presentation by faculty of patient findings, history and physical, differential diagnosis, treatment plan, etc.

Peer Teaching – learner-to-learner instruction for the mutual learning experience of both “teacher” and “learner”
